# Design of Three Residues Peptides against SARS-CoV-2 Infection

**DOI:** 10.3390/v14102103

**Published:** 2022-09-22

**Authors:** Carla Zannella, Annalisa Chianese, Giuseppe Greco, Biagio Santella, Giuseppe Squillaci, Alessandra Monti, Nunzianna Doti, Giuseppina Sanna, Aldo Manzin, Alessandra Morana, Anna De Filippis, Gianni D’Angelo, Francesco Palmieri, Gianluigi Franci, Massimiliano Galdiero

**Affiliations:** 1 Department of Experimental Medicine, Università degli Studi della Campania Luigi Vanvitelli, 80138 Naples, Italy; 2 Research Institute on Terrestrial Ecosystems, National Research Council (CNR), Via Pietro Castellino 111, 80131 Naples, Italy; 3 Institute of Biostructures and Bioimaging (IBB), National Research Council (CNR), 80134 Naples, Italy; 4 Department of Biomedical Sciences, University of Cagliari, Cittadella Universitaria, 09042 Cagliari, Italy; 5 Department of Computer Science, University of Salerno, Via Giovanni Paolo II, 132, 84084 Fisciano, Italy; 6 Department of Medicine, Surgery and Dentistry, “Scuola Medica Salernitana”, University of Salerno, 84081 Baronissi, Italy

**Keywords:** SARS-CoV-2, coronavirus, peptides, entry inhibitors, spike protein, docking

## Abstract

The continuous and rapid spread of the COVID-19 pandemic has emphasized the need to seek new therapeutic and prophylactic treatments. Peptide inhibitors are a valid alternative approach for the treatment of emerging viral infections, mainly due to their low toxicity and high efficiency. Recently, two small nucleotide signatures were identified in the genome of some members of the *Coronaviridae* family and many other human pathogens. In this study, we investigated whether the corresponding amino acid sequences of such nucleotide sequences could have effects on the viral infection of two representative human coronaviruses: HCoV-OC43 and SARS-CoV-2. Our results showed that the synthetic peptides analyzed inhibit the infection of both coronaviruses in a dose-dependent manner by binding the RBD of the Spike protein, as suggested by molecular docking and validated by biochemical studies. The peptides tested do not provide toxicity on cultured cells or human erythrocytes and are resistant to human serum proteases, indicating that they may be very promising antiviral peptides.

## 1. Introduction

The rapid and devastating spread of the Coronavirus disease 2019 (COVID-19) is posing a major challenge to human health. The severe acute respiratory syndrome coronavirus 2 (SARS-CoV-2) belongs to the *Coronaviridae* family, i.e., riboviruses characterized by a large RNA genome (26–32 kb) consisting of a single, positive and not segmented strand. Moving outward, the RNA is coated by the capsid with helical symmetry and, going further to the surface, by the viral envelope. Due to the presence of the spike protein (S) projecting outside the envelope, the virion appears as a spherical particle (60–140 nm in diameter) with a crown shape. Two coordinated events are mandatory for SARS-CoV-2 infection to occur [1]: (i) the S protein-mediated receptor binding, and (ii) the proteolytic cleavage of the S protein to induce virus-cell fusion. The S protein mediates SARS-CoV-2 entry by attaching to the human angiotensin-converting enzyme 2 (hACE2) and promoting the fusion with the host cell membrane [2]. The S protein is a class I fusion protein that forms a protruding homotrimeric spike consisting of two domains, S1 and S2 [3]. The amino-terminal S1 contains the receptor-binding domain (RBD) [4], meanwhile the carboxyl-terminal S2 represents the fusion motor domain [5]. The S protein is proteolytically processed: the host serine protease furin mediates the first cleavage at the S1/S2 boundary occurring after receptor binding and during the viral entry in late endosomes [2,6]. This modification is essential for driving the conformational change of S1 and the second cleavage in S2 subunit by the transmembrane serine protease 2 (TMPRSS2) [7]. Subsequently, the fusion peptide (FP) is exposed [8], thus S2 subunit moves to the stable post-fusion conformation in which a six-helix bundle (6HB) is formed and the fusion occurs [9]. Entry into the host cell is the first and crucial step in the virus lifecycle, therefore its inhibition is essential to stop the early phases of infection [10,11]. Recently, the research has been animated by the pressing need for new therapeutic alternatives for the treatment of SARS-CoV-2 infection. An essential step in the early stages of SARS-CoV-2 infection that can be targeted for designing possible antiviral drugs is represented by the activation of protein S by successive proteolytic cuts. Many potential inhibitors compete with the furin for binding to the cleavage site. A ketone-based peptide, named decanoyl-RVKR-chloromethylketone (CMK), has been previously demonstrated as a potent inhibitor of furin belonging to SARS-CoV-1 [12] and Middle East respiratory syndrome (MERS) [13], and it exhibited a marked activity in the inhibition of syncytia formation and virus infection. Recently, Cheng et al. showed that the treatment of Vero E6 cells expressing the S protein with CMK for 24 h blocked SARS-CoV-2 entry by causing an extensive reduction in the levels of processed S protein fragments [14]. A great variety of peptide drugs that targeted the fusion mechanism of class I viral proteins have been also investigated and optimized, such as for the Human immunodeficiency virus (HIV) [15,16,17,18], paramyxovirus [19,20,21] and Ebola virus [22]. Recently, Porotto et al. evidenced the strong antiviral potential of a lipopeptide derived from the C-terminal heptad repeat (HRC) domain of SARS-CoV-2 S protein. In detail, the 36-residue peptide (1168–1203) was linked to the Gly-Ser-Gly-Ser-Gly-Cys extension, used for the conjugation to cholesterol [23,24]. The lipopeptide was shown to potently inhibit S-mediated fusion with a 50% inhibitory concentration (IC_50_) at 10 nM [24]. In this scenario, peptides could represent powerful antiviral drugs, due to their high efficacy and specificity, and therefore constitute a very valid alternative option for the treatment of emerging viral pathogens. Usually, antiviral peptides are able to intervene in the initial stages of viral replication. They do this in the extracellular environment, without penetrating the cell membrane and, therefore, minimizing the potential damage to the host cell and side effects. Some of them are currently used in the clinic, such as the Enfuvirtide (Fuzeon), a peptide inhibitor approved by the US Food and Drug Administration (FDA) and the European Medicines Agency (EMA) in 2003 against HIV infections [25]. However, peptide inhibitors usually have a variety of drawbacks, among them generally, they have a very short half-life due to the ease of cleavage by serum proteases. In addition to the rapid clearance in vivo, as their size increases, peptides can become not easily soluble and can be associated with high production and manufacturing costs. The present study was focused on two small peptides, each consisting of only three amino acid residues (TLH and VFI), deriving from two recurrent nucleotide signatures recently identified in many pathogenic microorganisms (ACATTACAC and GTGTTTATT) [26], including in different members of *Coronaviridae* family. These two sequences are not coding regions in the genome of SARS-CoV-2, as they are not in the coding reading frame of S gene. However, in the light of this significant evidence [24] and of the increasing importance of small open-reading frame (sORF)-encoded peptides (SEPs) [27,28,29,30], recurrent especially in microorganisms [31,32,33], we investigated whether the translation of these highly conserved base-pairing regions could give compounds with antiviral activity. We found that the two tripeptides are able to inhibit betacoronavirus entry mechanism by binding the RBD of SARS-CoV-2 Spike protein, as suggested by molecular docking and validated by biochemical studies. Interestingly, peptides also inhibit the entry mechanism of a second betacoronavirus, i.e., HCoV-OC43, but they are not active against the alphacoronavirus HCoV-229E. In addition, the tripeptides had a very low toxicity profile and a good half-life in serum, suggesting their potential use as template for further development of antiviral drugs against SARS-CoV-2 infection.

## 2. Materials and Methods

### 2.1. Genome Alignment

Several viral genomes were analyzed in order to search the two recurrent nucleotide sequences (ACATTACAC and GTGTTTATT). The genomic sequences were downloaded from the National Center for Biotechnology Information (NCBI) database and included human coronavirus HCoV-229E (NC_002645), HCoV-OC43 (MW532119), SARS-CoV-2 (NC_045512), SARS-CoV-1 (NC_004718), MERS (NC_019843), Herpes simplex virus type 1 (HSV-1, NC_001806), Enterovirus A-71 (EV-A71, MT241239), HIV-1 (HIVMVP5180), Yellow fever virus (YFV, JX503529), West Nile virus, (WNV, HM488124), and Dengue virus type 2 (DENV-2, KF360005) (Table 1).

### 2.2. Peptides Synthesis

Protected amino acids, coupling agents (HATU, Oxyma) and Fmoc-Rink Amide AM resin used for peptide synthesis were purchased from IRIS Biotech GmbH (Marktrewitz, Germany). Solvents, including acetonitrile (CH_3_CN), dimethylformamide (DMF), trifluoroacetic acid (TFA), sym-collidine, diisopropylethylamine (DIPEA), piperidine, were from Merck (Milan, Italy). Peptides were synthesized on solid phase on Rink-Amide MBHA resin (loading 0.4–0.8 mmol/g) following the Fmoc strategy and using HATU-collidine as coupling reagents [34,35]. A mixture composed of trifluoroacetic acid (TFA)/tri-isopropylsilane (TIS)/water (95:2.5:2.5, *v*/*v*/*v*), which was stirred for 3 h at room temperature, was used for the peptide cleavage. Then peptides were precipitated by cold diethyl ether and the resulting pellets were resuspended in a mixture of H_2_O/CH_3_CN (75:25 *v*/*v*) and lyophilized. Crude peptides were purified by reverse-phase HPLC (RP-HPLC) on a WATERS 2545 preparative system (Waters, Milan) equipped with a WATERS 2489 UV/Vis detector. Purification step was performed at 15 mL/min using a Jupiter C18 (5 μm, 150 mm × 21.2 mm ID) column applying a linear gradient of 0.1% TFA in CH_3_CN from 5% to 70% over 15 min, monitoring the absorbance at 214 nm. The identity of peptides was assessed by liquid chromatography-mass spectrometry analysis (LC-MS) using an ESI-TOF-MS Agilent 1290 Infinity LC System coupled with a photodiode array (PDA) detector and a 6230 time-of-flight MS detector, along with a binary solvent pump degasser, a column heater and an autosampler (Agilent Technologies, Cernusco sul Naviglio, Italy). LC-MS characterization of peptides was performed using a C18 Waters xBridge column (3 μm, 4.6 mm × 5.0 mm), applying a linear gradient of CH_3_CN/ 0.05% TFA in H_2_O/0.05% TFA from 5 to 70% in 15 min, at a flow rate of 0.2 mL/min. Peptide spectra are provided as Supplementary Figure S1.

### 2.3. Peptide Stability

Serum from a healthy donor was first equilibrated at 37 °C for 5 min in a thermostatic bath (BM 12, Falcinstruments, Treviglio, Italy). Then, each tripeptide was incubated at 1 mM and 100 μL serum aliquot was withdrawn at each time-point (0, 1, 2, 6, 12, 24 h). A control blank (not treated serum) was used as negative control. Each experiment was performed in duplicate. Samples were then vortexed and centrifuged (15 min, 20,000× *g*, 4 °C). The supernatants were ultra-filtered through an Amicon Ultra Centrifugal Filter Unit (Cut-off 3 KDa), in centrifuge at 4 °C, 10,000× *g*). The filtrates were then used for HPLC analyses. TLH and VFI peptides were identified and quantified by Reverse Phase High-Performance Liquid Chromatography (RP-HPLC) using a Dionex Ultimate 3000^®^ HPLC system equipped with quaternary pump and Ultimate 3000^®^ Diode Array Detector. The samples were injected in a Luna C18 (2) column (250 mm × 4.6 mm, 5.0 μm, Phenomenex, Torrance, CA, USA) with SecurityGuard™ pre-column containing a C18 cartridge. The elution method was as follows: flow rate fixed at 800 μL/min; solvent A: 0.1% formic acid in degassed ultrapure water; solvent B: 0.1% formic acid in acetonitrile; from min 0 to min 6 stable flow at 0.5% of B, from min 6 to min 55 a linear gradient reaching 95% of B followed by 12 min of maintenance. The detector was set at 210 nm with the spectra recording function active. Tripeptides were identified by comparing the retention time and the absorption spectra of pure peptide solutions. The peak areas corresponding to the peptides were calculated using the Dionex^®^ Chromeleon^®^ peaks integration tool and linearized by logarithm. The resulting equations of the straight line passing through the points were the following:

y = −0.0652x + 1.7094 (R2 = 0.99), for the TLH peptide(1)

y = −0.0249x + 2.5659 (R2 = 0.99), for the VFI peptide(2)

The half-life of the tripeptides was calculated by applying the respective equations.

### 2.4. Cells and Viruses

Baby hamster kidney (BHK-21) [ATCC CCL 10 (C-13), *Mesocricetus auratus*] and monkey kidney (Vero-76) [ATCC CRL 1587, *Cercopithecus aethiops*] were purchased from American Type Culture Collection (ATCC, Manassas, VA, USA) and grown in DMEM (Microtech, Naples, Italy) additioned with 10% FBS (Microgem, Naples, Italy) and 1X penicillin/streptomycin solution (Himedia, Mumbai, India). CD4+ human T-cells containing an integrated HTLV-1 genome (MT-4) were obtained from NIH AIDS Research and Reference Reagent Program (Rockville, MD, USA) and cultured in Roswell Park Memorial Institute (RPMI) 1640 (Himedia) supplemented with FBS and antibiotic solution. Cell cultures were checked periodically for the absence of mycoplasma contamination with MycoFluor Kit (ThermoFisher, Waltham, MA, USA). Viruses were purchased from ATCC and included: (i) *Coronaviridae*: human coronavirus strain HCoV-229E (ATCC VR-740), HCoV-OC43 (ATCC VR-1558) and SARS-CoV-2 [clinical isolate]; (ii) *Picornaviridae*: EV-A71 strain BrCr (ATCC VR-1775); (iii) *Flaviviridae*: YFV [strain 17-D vaccine (Stamaril Pasteur J07B01)], WNV [clinical isolate], DENV-2 [clinical isolate]; (iv) HIV-1 IIIB laboratory strain was obtained from the supernatant of the persistently infected H9/IIIB cells (NIH 1983); (v) HSV-1 strain KOS (ATCC VR-1493). Viruses were maintained in our laboratory and propagated in appropriate cell lines. The viruses were stored in small aliquots at −80 °C until use. All experimental work involving viruses was performed in an appropriate biosafety level containment laboratory.

### 2.5. Cytotoxicity Assay

MT-4 cells (4 × 10^5^ cell/mL), BHK-21 cells (1 × 10^6^ cell/mL) and Vero-76 (2 × 10^5^ cell/mL) were seeded in 96-well plates and then incubated at 37 °C in a humidified atmosphere. The next day, cell suspension or cell monolayers were incubated in the absence or presence of serial dilutions (in the range 1 ÷ 200 μM) of each tripeptide. The test media used for the cytotoxicity assay were the same used for cell cultures. Cell viability was determined after 24 h by the 3-(4,5-dimethylthiazol-2-yl)-2,5-diphenyltetrazolium bromide (MTT) method and was equal to:

[1 − (Abs of treated samples − Abs of blank/Abs of control samples − Abs of blank)] × 100(3)

where Abs of blank and control samples refer to the absorbance of solvent and not treated cells, respectively.

### 2.6. Hemolysis Assay

Hemolysis was measured on human red blood cells (taken from healthy volunteers). Briefly, a suspension of human erythrocytes was mixed with two-fold serial dilutions of each tripeptide and this mixture was then incubated for 1 h at 37 °C. Subsequently, the samples were centrifuged for 5 min at 500 g and the absorbance of the supernatant was measured at 415 nm using the microplate reader.

### 2.7. Antiviral Testing

To understand whether the tripeptides were able to inhibit coronavirus infectivity and, specifically, their mode of action, four different assays were performed. The difference between the four schemes of treatment is the timing of the addition of tripeptides [36].

(a) Co-treatment test. This is a screening assay to point out the tripeptides’ activity as antiviral agents. Then, each tripeptide was added to the cell monolayer (1–200 μM) at the same time as viral infection at a multiplicity of infection (MOI) of 0.1 plaque forming unit (pfu)/cell for 2 h at 37 °C.
(b) Virus pre-treatment. This test is useful for evaluating whether tripeptides can act directly on the viral particles. Each peptide was added to the virus (1 × 10^4^ pfu/mL) and incubated for 1 h at 37 °C. After incubation, the mixture (virus/peptide) was diluted on cells and incubated for 2 supplementary hours, so that the peptide reaches a non-active concentration and the virus was at a MOI of 0.01 pfu/cell.
(c) Cell pre-treatment. To assess whether tripeptides could interact with the target cell, preventing the subsequent binding to the virus surface. Cells were pre-cooled at 4 °C for 30 min and, subsequently, the peptide was added and incubated for 1 h at 4 °C. Then virus was added to a MOI of 0.1 pfu/mL for 2 h at 37 °C.
(d) Post-treatment. The assay allows the assessment of the tripeptides’ ability to interfere with the viral replication. Cells were incubated with virus (MOI 0.1 pfu/mL) for 2 h at 37 °C, after that the peptide was added and incubated on the cells for two different time lapses, i.e., 1 h and 24 h.

For all the above treatments, Vero cells were plated in 12-wells to reach the confluence of 5 × 10^5^ cell/mL on the day of infection. After 2 h of infection, the non-penetrated viruses were deactivated by the citrate buffer (pH 3.0); infected cells were covered with a fresh culture medium supplemented with carboxymethylcellulose (CMC, Sigma-Aldrich, St. Louis, MO, USA) and incubated for 48 h. Then, the monolayers were fixed, stained with crystal-violet solution (Sigma-Aldrich, St. Louis, MO, USA) and the plaques were counted. All experiments were performed in triplicate. The inhibition rate of the infectivity was evaluated by plaque assay comparing the number of plaques obtained in the wells treated with the peptides to the plaques counted in the negative control (cells infected with virus, without peptide). Concentrations resulting in 50% inhibition (EC50) were determined by linear regression analysis. The activity of the tripeptides against HIV-1 _IIIB_ laboratory strain, DENV-2, WNV, YFV and EV-A71 was based on inhibition of virus-induced cytopathogenicity in appropriate cell lines, infected at a MOI of 0.01. Cell viability was determined by the MTT method [37]. For HSV-1, plaque assays were carried out with the same approaches described above.

### 2.8. Statistical Analysis

All tests were performed in triplicate and expressed as mean ± Standard Deviation (SD) calculated using GraphPad Prism (version 5). Statistical differences were evaluated via One-way ANOVA followed Dunnett’s multiple comparisons test and a value of *p*  ≤  0.05 was considered significant.

### 2.9. Molecular Docking

The spike protein structure (PDB 6XM4) was downloaded from the protein database (https://www.rcsb.org/, accessed on 27 July 2022). Water molecules were removed from the structure using the PyMOL system. The chemical structures of the two tripeptides were designed in ChemBio3D Ultra 13.0 and converted to secondary structure by energy minimization steps. Molecular docking was implemented by using HPEPDOCK web server with its default parameters. We provided the processed structure protein as the receptor input, and HPEPDOCK generates tridimensional structure models for a given peptide sequence using the implemented MODPEP program. One hundred docking models (poses), classified according to binding energies, were generated for each docking experiment. The selection of the docking pose (from the first 10 poses that had the best binding energies) was based on a comparison of the docked peptides in the Spike structure with that of each tripeptide resolved structure and the binding energy values. Subsequently, to improve the resolution of the tripeptide docking region with SARS-CoV-2 fusion protein, we selected the interaction region with PyMOL software and solved it with iGEMDOCK v2.1 software, which was implemented with a generic evolutionary algorithm to perform automated molecular docking experiments.

### 2.10. Octet Biolayer Interferometry (BLI)

An Octet^®^ Red 96 system^®^ (ForteBio, Fremont, CA, USA) was used to detect interactions between the His-tagged Spike RBD protein (Elabscience Biotechnology, Biomedical Park, Wuhan, China; Cat. No.: PKSH032068) and peptides using Octet His2 biosensors according to the standard instructions with minor modifications. The running volume for buffers or samples was 100 µL. The rpm and the temperature of the plate were kept at 600 rpm and 25 °C, respectively, for the entire assay. Biosensors were hydrated with PBST (10 mM phosphate, 150 mM NaCl, 0.05% Tween 20, pH 7.4) for 10 min before each assay. In each experiment, biosensors were first equilibrated with PBST for 120 s to detect the baseline. Then, the association of the His-tagged Spike RBD protein alone (0.2 µM), of the free peptides in solution (160 µM) and of the protein (0.2 µM) pre-mixed to each peptide tested at several concentrations (40, 80 and 160 µM) to the biosensor, was recorded in parallel until an equilibrium was reached (1200 s). Afterwards, the dissociation step was performed in PBST for 1200 s. All experiments were performed in duplicates. Biosensors were discarded after each measurement.

## 3. Results

### 3.1. Design of Tripeptides

In the present study we evaluated the presence of two nucleotide sequences (ACATTACAC and GTGTTTATT), identified in many pathogenic microorganisms, in the sequences of viral surface proteins involved in virus fusion and entry into the host cell. In detail we focused on the genomic regions coding the spike protein of SARS-CoV-2, HCoV-229E, HCoV-OC43, SARS-CoV-1 and MERS, glycoprotein B of HSV-1, VP1 of EA-71, gp120 of HIV-1 and Flaviviridae glycoprotein E, as reported in Table 1. Their presence/absence was annotated by indicating the starting position in the genome: ACATTACAC and GTGTTTATT were located at position 25,372 and 23,463, respectively, in the SARS-CoV-2 genome; GTGTTTATT was present in the HCoV-OC43 genome at 24,582, and, in contrast, the sequence ACATTACAC was conserved in SARS-CoV-1 and MERS RNA at positions 25,247 and 25,227, respectively. The nucleotide sequences were not identified in any other viral genomes selected for the present study, e.g., in HCoV-229E, HSV-1, EA-71, HIV-1, YFV, WNV and DENV-2. In Supplementary Figure S2, the chemical and structural conformations of the two peptides derived from ACATTACAC and GTGTTTATT sequences are reported.

### 3.2. Peptides Toxicity

**Figure 1:**
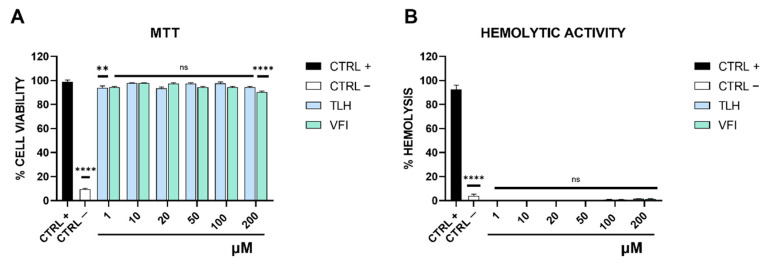
Tripeptides toxicity in vitro cultures (**A**) and human erythrocytes (**B**). CTRL+ refers to positive control (untreated cells for MTT assay, and Triton X-100 20% for the hemolysis test) and CTRL— indicates the negative control (DMSO-treated cells for MTT assay, and PBS-treated cells for the hemolysis test); **** *p*  ≤  0.0001; ** *p*  ≤  0.01; ns: non-significant.

Firstly, we evaluated the potential toxicity of the two tripeptides. To do this, we used two different methods: (i) the metabolic MTT assay and (ii) the haemolytic assay. As reported in Figure 1A, neither of the tripeptides were toxic on Vero-76 cells in all tested concentrations (from 1 to 200 μM) after 24 h (h).

Similar results were obtained testing peptides on different cellular models, such as the human T-cell line (MT-4) and hamster kidney fibroblasts (BHK-21) (Supplementary Table S1). Moreover, no haemolytic toxicity was observed in human erythrocytes treated with tripeptides in the same concentrations described above for 1 h at 37 °C (Figure 1B).

### 3.3. Antiviral Activity

To explore the potential antiviral activity of tripeptides against coronaviruses (HCoV-229E, HCoV-OC43 and SARS-CoV-2), we performed different antiviral assays following four experimental schemes: co-treatment, virus pre-treatment, cell pre-treatment and post-treatment. In the co-treatment assay, the virus and the peptide were incubated together on the cells for 1 h; in the virus pre-treatment the virus was first treated with the peptide for 1 h and then added to Vero cells. On the contrary, in the cell pre-treatment, cells were pre-treated with peptide for 1 h and then infected with the virus. Finally, in the post-treatment test, cells were first infected for 1 h, and then subsequently incubated with the peptide for another 1 h incubation. Tripeptides were able to interfere considerably with the viral infection.

**Figure 2:**
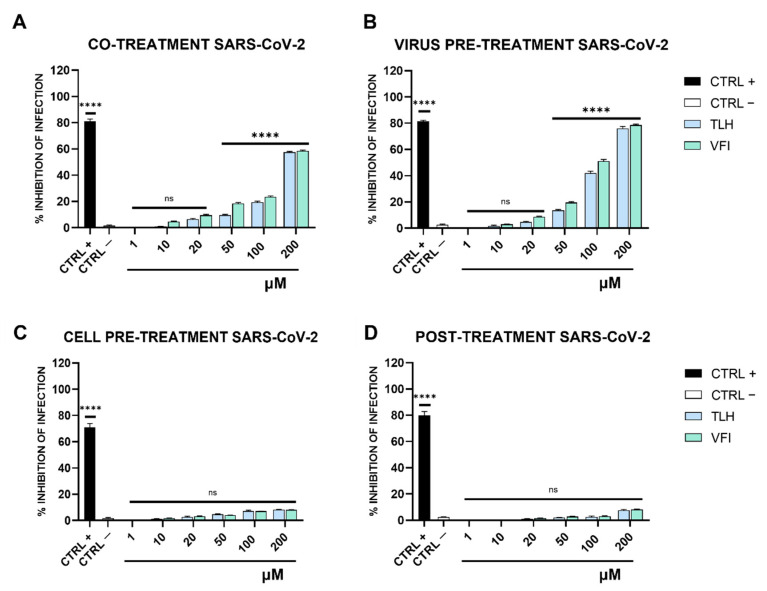
Antiviral activity of tripeptides against SARS-CoV-2. (**A**) co-treatment; (**B**) virus pre-treatment; (**C**) cell pre-treatment; (**D**) post-treatment. CTRL+ refers to positive control (ivermectin 12 μM) and CTRL—indicates the negative control (only infected cells); **** *p*  ≤  0.0001; ns: non-significant.

As depicted in Figure 2A,B, the tripeptides were active against SARS-CoV-2 with a very similar efficacy both in co-treatment and virus pre-treatment assays.

Both peptides reduced SARS-CoV-2 infection up to 60% at 200 μM when they were added simultaneously to the virus on the target cells (co-treatment, Figure 2A). An increased and comparable inhibitory effect was observed in virus pre-treatment assay (Figure 2B). Indeed, at 200 µM they blocked the infection up to 80%. Otherwise, no significant effects were observed, either when the peptides were added to cells (cell pre-treatment, Figure 2C) or after the virus infection (post-treatment, Figure 2D).

**Figure 3:**
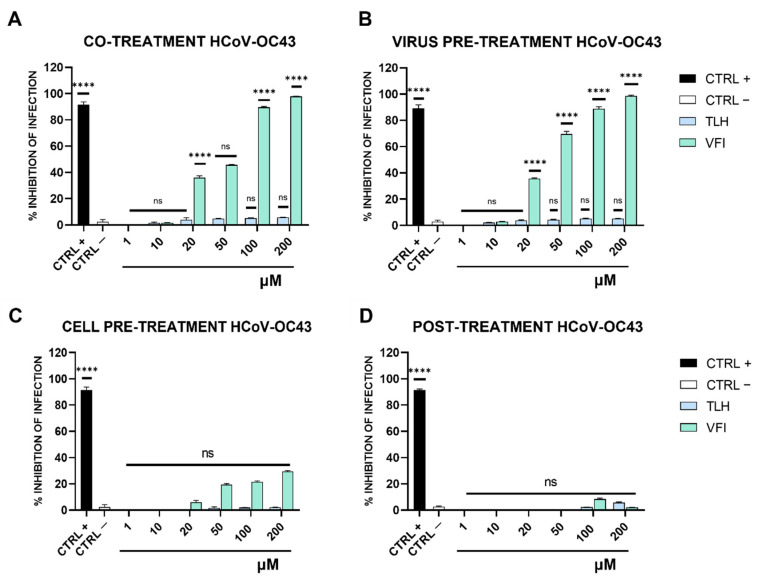
Antiviral activity of tripeptides against human coronavirus OC43. (**A**) co-treatment; (**B**) virus pre-treatment; (**C**) cell pre-treatment; (**D**) post-treatment. CTRL+ refers to positive control (ivermectin 12 μM) and CTRL—indicates the negative control (only infected cells); **** *p*  ≤  0.0001; ns: non-significant.

In contrast to what was observed for SARS-CoV-2, only the tripeptide VFI was able to inhibit HCoV-OC43 infection in co-treatment and virus pre-treatment assays in a dose dependent manner (Figure 3A,B). In co-treatment assay, the peptide VFI completely blocked HCoV-OC43 infection at the highest concentration of 200 μM and reduced the viral replication by 80% and 45% at concentrations of 100 and 50 μM, respectively. In the virus pre-treatment assay, its antiviral ability appeared enhanced, by reducing considerably the viral infection by 70% at 50 μM.

Similar to what was observed for SARS-CoV-2, no significant effects were observed either when the peptide VFI was added to cells (cell pre-treatment, Figure 3C) or after the virus infection (post-treatment, Figure 3D). TLH did not show any inhibitory effect against HCoV-OC43 infection in the same treatments and experimental conditions (Figure 3).

Surprisingly, neither peptide showed any antiviral activity against the alphacoronavirus HCoV-229E (Supplementary Figure S5) and the other tested viruses (Supplementary Table S1). Altogether, results highlight that not only is there a tripeptide-driven mechanism able to block specifically betacoronavirus infection, but also that tripeptides can act specifically on the virus particles, preventing them from interacting with the host cell.

### 3.4. In Silico Analysis

**Figure 4:**
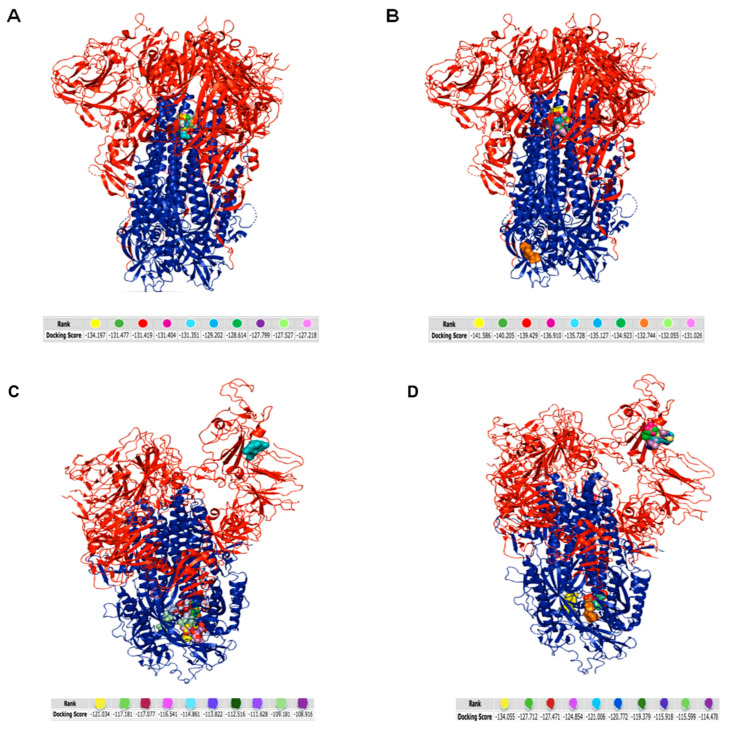
Molecular simulation of tripeptides interacting with HCoV-OC43 and SARS-CoV-2 spike protein obtained by HPEPDOCK server: (**A**) TLH/HCoV-OC43 spike protein; (**B**) VFI/HCoV-OC43 spike protein; (**C**) TLH/SARS-CoV-2 spike protein; (**D**) VFI/SARS-CoV-2 spike protein. The red color indicates the S1 subunit and the blue color the S2 subunit of the spike protein. The different color code of peptides, represented as balls, refers to the different binding free energy.

On the basis of the peculiar inhibitory activity showed by the tripeptides tested, we investigated the potential interaction between tripeptides and viruses by docking studies between each peptide and the viral protein deputed to the attachment and fusion with the cell membrane (Figure 4 and Supplementary Figure S3). The docking predictions were obtained by the HPEPDOCK server [38] and showed interesting patterns for both HCoV-OC43 and SARS-CoV-2.

The tripeptide TLH could bind to the HCoV-OC43 spike protein in internal and hidden sites (Figure 4A); by contrast, the tripeptide VFI (Figure 4B) docked within the viral protein at the same internal pocket of TLH, but, additionally, with a S2-located and exposed site with a docking energy score of −132.744. The greater accessibility of the site linked by VFI on the HCoV-OC43 spike protein justified its exclusive antiviral activity described in the Figure 2A,B. However, the observation that the tripeptide TLH was only able to recognize and bind internal sites of the spike could explain its absent activity in inhibiting HCoV-OC43 infection, as shown in Figure 2A,B. Moving to SARS-CoV-2, the tripeptides interacted very similarly between them with the spike protein, as demonstrated by the in silico simulations (Figure 4C,D) and as already reported in plaque assays in Figure 3A,B. Data suggested that both the tripeptides bind to the S2 subunit and the docking energy score was −121.034 and −134.055 for the tripeptides TLH and VFI, respectively. However, they were also able to dock with the S1 subunit with a very different energy score (−109.181 for TLH and in the range of values from −127.712 for VFI), highlighting their potential ability to block both viral attachment and fusion steps to the target cell. As a control, we carried out the same analysis with an unrelated peptide AAA (Supplementary Figure S4). Docking the peptide on SARS-CoV-2 spike protein, we observed that the binding energy was very low, evidencing that any interaction was highly probable.

### 3.5. Analysis of the Interaction between Tripeptides and SARS-CoV-2 Spike Protein

**Figure 5:**
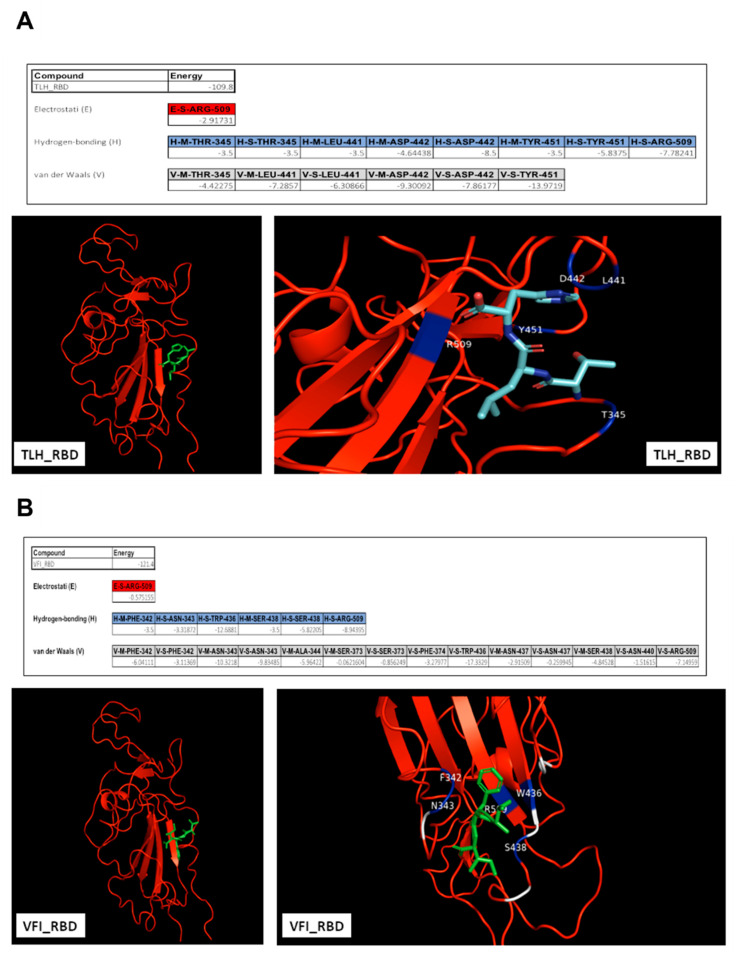
Interacting amino acids at the interface of SARS-CoV-2 RBD (PDB 6LZG). (**A**) TLH; (**B**) VFI. Electrostatic interactions, hydrogen bonds and van der Waals forces are indicated as E, H and V, respectively.

To further characterize the interaction between peptides and the SARS-CoV-2 S protein, we analyzed the interaction forces and identified the putative residues of the protein involved in the binding with tripeptides. Then, each tripeptide was docked against SARS-CoV-2 RBD (PDB 6XM4) using the HPEPDOCK server (Figure 5).

**Figure 6:**
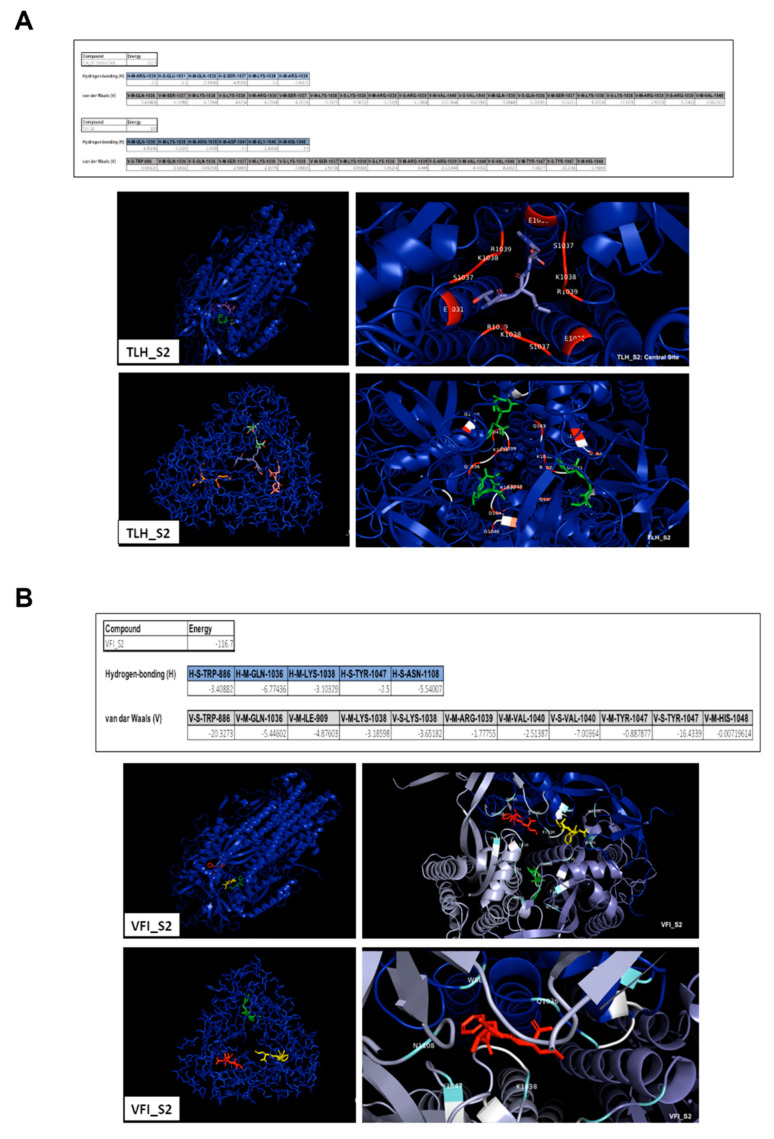
Interacting amino acids at the interface of SARS-CoV-2 S2 subunit. (**A**) TLH; (**B**) VFI. Electrostatic interactions, hydrogen bonds and van der Waals forces are indicated as E, H and V, respectively.

Free energy calculations of the peptides interacting with RBD demonstrated that van der Waals forces, electrostatic interactions and hydrogen bonds could be the driving forces stabilizing the binding. Arginine in position 509 (R509) could be crucial for both the complexes for the establishment of electrostatic interactions. The tripeptide TLH could bind to the SARS-CoV-2 RBD via the interacting residues threonine 345 (T345), leucine 441 (L441), aspartic acid 442 (D442), tyrosine 451 (Y451) and R509, involved in hydrogen bonds and also in van der Waals forces. VFI peptide could interact with RBD by other residues; in detail, phenylalanine 342 (P342), asparagine 343 (N343), alanine 344 (A344), serine 373 (S373), F374, tryptophan 436 (W436), N437, S438, N440 and R509 could participate in the van der Waals forces, and P342, N343, W436, S438 and R509 could also be involved in hydrogen bonds. We then evidenced that both the peptides could interact with the hydrophobic side of the β-sheet core of RBD, defined by W436, F374 and the side chain of R509, and L441 on the other side, engaging hydrophobic contacts. It has been already widely reported that residues from 474 to 504 in the spike protein are critical for ACE2 binding [39]. The occupancy of these pockets could therefore be essential for inhibiting the subsequent hACE2 recognition and binding. A similar analysis was also carried out for the putative interaction between peptides and the SARS-CoV-2 S2, indicating several binding sites even in the fusion machinery of the virus (Figure 6).

Free energy calculations indicated that the interactions between peptides and the spike S2 subunit were stabilized by hydrogen bonds and van der Waals forces. Interestingly, both the peptides could interact with sites containing important clusters of highly conserved residues. The predictions revealed that the peptide TLH could bind to S2 via the interacting residues W886, glutamine 1036 (Q1036), S1037, lysine 1038 (K1038), R1039, valine 1040 (V1040), Y1047 and histidine 1048 (H1048), all involved in the formation of van der Waals interactions. Most of them were also able to participate in the establishment of hydrogen bonds and were predicted to be involved in the interactions existing between the other tripeptide VFI and the SARS-CoV-2 S2 subunit too, in particular residues from Q1036 to H1048. The sequence conservation of key residues highlights an important potential advantage for developing RBD and S2 targeted fusion inhibitors. Some of these residues have also been identified in other studies [40,41,42,43] as key residues for the interaction with ACE2 (amino acids present in RBD), or as involved in the fusion with the host cell (amino acids present in the S2 subunit).

### 3.6. Direct Binding Assays between Tripeptides and SARS-CoV-2 Spike Protein

**Figure 7:**
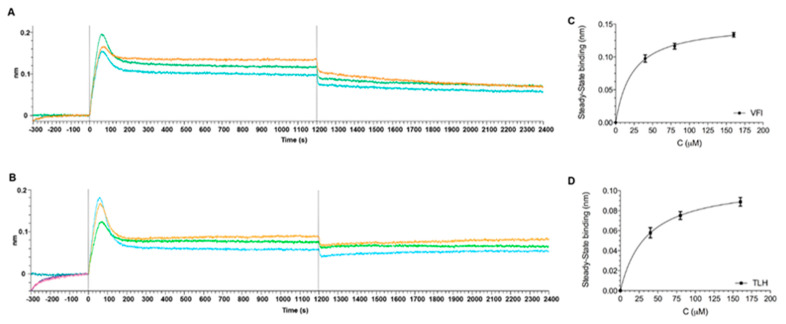
BLI interaction analysis. RBD (0.2 µM) (**A**,**B**) either alone in solution or mixed with VFI (**A**) and TLH (**B**) peptides, tested at different concentrations (40 µM, blue lines; 80 µM, green lines and 160 μM, orange lines). BLI interaction was performed at 25 °C in PBST (10 mM phosphate, 150 mM NaCl, 0.05% Tween 20, pH 7.4). The corresponding plots (**C**,**D**) of steady-state binding from the end of the association phases (nm), after the subtraction of RBD signal, against analyte concentration were used to calculate the steady-state affinity by nonlinear regression analysis using GraphPad 5 software.

The interaction of peptides against the Spike protein of SARS-CoV-2, was also assessed in real-time by the Biolayer Interferometry (BLI) technology using the Octet^®^ Red 96 system^®^, using the His-tagged Spike RBD protein (hereafter RBD). Given the very low MW of the peptides (~300 Da), none of them could be detected through direct binding on the biosensors coated with the protein (26 kDa), as their binding signals were detected within the noise of the instrument. Therefore, we measured the effect of each peptide by pre-mixing it, at different concentrations (40, 80 and 160 µM), with the protein and comparing the result with the binding of the protein alone on the sensor chip. For these experiments Octet His2 biosensors, with pre-immobilized anti-HIS antibody, were used. Thus, the RBD only (0.2 μM), TLH or VFI alone (160 μM), and the RBD-TLH or RBD-VFI pre-mixed analytes, at different molar ratios, were incubated over the biosensors. Raw data showed that while TLH and VFI peptides failed to bind to the biosensor surface even at the highest tested concentration (160 μM), the pre-mixed RBD-TLH and RBD-VFI resulted in an increased binding response in a concentration-dependent manner over the RBD alone (Supplementary Figure S6). Therefore, the maximum wavelength shifts reached after equilibration of the reactions, of signals left after background (signal of protein alone) subtraction were determined and plotted against the corresponding peptide concentrations to determine the KD values (Figure 7).

Results obtained showed that peptides bind RBD, in dose-dependent manner, showing an apparent affinity constant in the micromolar range (KD = 28.4 ± 4.4 and 22.8 ± 0.1 µM, respectively for VFI and TLH). Notably, two other synthetic peptides (FIV and AAA), used as controls, either displayed no binding or had significantly reduced affinity in the same experimental conditions mentioned above (Supplementary Figure S7). The wavelength shift obtained in these cases compared to the RBD protein were very low, indicative of a weak or non-specific interaction. Fitting the data did not provide representative KD values.

### 3.7. Stability of Tripeptides in Human Serum

**Figure 8:**
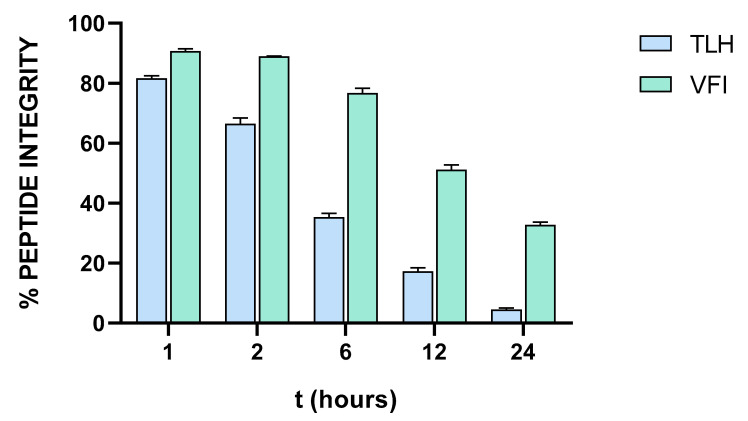
Time-course of the incubation of tripeptides in human serum.

Proteolytic instability is a critical limitation for peptide-based products as potential drugs. To analyze the tripeptides half-life, they were incubated with 50% (*v*/*v*) human serum for different times and then analyzed by liquid chromatography/mass spectrometry for the evaluation of peptides integrity (Figure 8).

Peptide TLH was more quickly degraded than VFI. Indeed, the first one exhibited a half-life of 4.36 h, while VFI is more stable in human serum, showing a 50% of peptide integrity after 12.44 h. According to the small size of the tripeptides, it is reasonable to assume that they are recognized and cleaved by oligopeptidases, which are cell-surface enzymes involved in the regulation of the biological activity of small bioactive peptides [44]. The peptide TLH is more efficiently cleaved since it consists of the leucine residue that is easily recognized by aminopeptidases (aminopeptidase N); on the other hand, VFI exhibits only binding sites for ectopeptidases (neprilysin) cleaving mainly longer amino acid sequences [45].

## 4. Discussion

The advent of SARS-CoV-2 vaccines has significantly slowed down the spread of the virus, but there is still no cure to treat the infection once it is contracted, and there is an urgent request for innovative prophylactic approaches. Peptides represent a valid and safe alternative for treating viral infections [46] but, however, there are still few studies investigating the role of peptides in SARS-CoV-2 infection [47,48,49,50]. Many peptides currently analyzed for their anti-SARS-CoV-2 effect are repurposing peptides. For example, aprotinin is a very long peptide (58 amino acid residues) already well-known as a transmembrane serine protease 2 (TMPRSS2) inhibitor influencing negatively influenza A and B viruses [51,52] replication. Several antimicrobial peptides (AMPs) have also been tested for their anti-SARS-CoV-2 activity. Recently, we described the broad-spectrum antiviral potential of the amphibian AMP temporin L and its analogs [36]. Temporin L has been tested against a wide panel of enveloped and naked DNA and RNA viruses, including SARS-CoV-2, and its effect is specifically addressed to early stages of viral infection. Furthermore, we modified the peptide by lipidation and demonstrated a highly reduced cytotoxicity with improved antiviral effect. Lipidation is a widely useful strategy to potentiate the antiviral activity of peptides and also reduce their toxicity profile. The addition of cholesterol and fatty acid moieties has a dual role: (i) the lipid tail helps the peptide to insert in the lipid membranes and (ii) the peptide self-aggregates into micelles concentrating its presence at the action site. Another AMP widely studied was the so-called peptide P9 derived from the mouse-β-defensin-4, endowed with inhibitory abilities against several respiratory viruses, such as SARS-CoV-1, MERS and influenza virus [53,54,55,56,57]. The majority of repurposed peptides is based on the viral fusion protein spike. In detail, they are HR-derived peptides able to form 6HB-like structures which assemble together with the intermediate extended form of the fusion protein trimer, and interrupt the structural rearrangement of S. These peptides are therefore fusion inhibitors whose mechanism of action has been largely studied also for other class I fusion proteins [58,59,60,61,62,63,64,65]. Recently, Porotto et al. described the strong antiviral potential of a lipopeptide derived from the SARS-CoV-2 S protein HRC domain [24]. The peptide, spanning from the residue 1168 to 1203, was first conjugated at the C-terminal end with a segment offering the cysteine residue; then, the 42 amino acids produced peptide was attached to a cholesterol moieties, displaying very strong potentialities in: (i) inhibiting cell-cell fusion by the S protein; (ii) reducing live SARS-CoV-2 infection in Vero E6 cell type; (iii) blocking the infection of a broad range of coronaviruses, including SARS-CoV-1 and MERS; (iv) preventing SARS-CoV-2 spread also in ex vivo model, such as human airway epithelial (HAE) cells. The small therapeutic window represents one of the main problems in peptide medical use. Indeed, generally there is a toxicity that increases with the structure complexity and length of the peptides. When the length of the peptides was reduced, the antimicrobial activity was lost. Han et al. evaluated five ACE2-derived peptides spanning in the region from the amino acid 22 to 57 of the cellular receptor [66]. In detail, the shorter peptides (P1: 22–31; P2: 30–38; P3: 33–41) did not exhibit any relevant anti-SARS-CoV-1 activity, even at the highest concentration of 100 μM; by contrast, the longer peptides (P4: 22–44; P5: 22–57) were significantly active against SARS-CoV-1 pseudovirus infection, as indicated by the low IC_50_ of 50 μM and 6 μM, respectively. Here, we focused our attention on two very small peptides deriving from an evaluation of an algorithm recently published [26]. D’Angelo et al. provided data for identifying recurrent nucleotide substrings starting from 5000 different SARS-CoV-2 genomes. Their results highlighted the existence of specific patterns constituted by the two substrings ACATTACAC and GTGTTTATT, present not only in SARS-CoV-2 genomes, but also in other viruses, such as SARS-CoV-1, MERS, HCoV-OC43, Nipah virus, and, also in the bacterium genus of *Streptococcus*. Starting from these two signatures, we synthesized the two corresponding peptides, i.d. TLH and VFI, each consisting of only three amino acid residues. Their small size does not allow their structuring, but we observed that both are capable of interacting with the SARS-CoV-2 spike through in silico models. Our results demonstrated that tripeptides can occupy pockets inside S1 and S2 domains, potentially blocking all downstream events and the entire SARS-CoV-2 replication cycle. We also analyzed their ability to interact with a very similar spike protein (30% identity and 42% similarity), i.e., that of HCoV-OC43, via in silico approach, observing that only the VFI peptide can bind to an external S1 site. These predictions were corroborated by in vitro assays evidencing that tripeptides were highly effective in the inhibition of SARS-CoV-2 infection. Their inhibitory activities, more prominent for the VFI peptide, were realized directly on the viral particles, probably by blocking the events driven by the spike protein. A very comparable effect was noted against HCoV-OC43, where only the VFI peptide exhibited a marked efficacy in inhibiting the early phases of viral infection. The peptides’ cytotoxicity profiles were very encouraging since they did not show any toxicity either in vitro cell cultures or on human erythrocytes. Altogether, these data open a potential clinical application of the two tripeptides. However, it is important to take into account the other main drawbacks of peptides, i.e., absorption, distribution, metabolism, and excretion, which limit their clinical translation [67,68,69]. The principal obstacle in the application of peptides for drug delivery is their susceptibility to proteolysis in the blood. Here, evaluating the stability of the two tripeptides in human serum, it’s clear that the peptide VFI had a longer half-life, since it appeared still intact after 12.44 h in the human serum.

## 5. Conclusions

We have demonstrated here for the first time that two small peptides are valid options in the prevention and treatment of SARS-CoV-2 infection. They were designed on two small nucleotide signatures in SARS-CoV-2 spike gene found also in the genome of other human pathogens. Peptides showed a very interesting antiviral effect against SARS-CoV-2 and HCoV-OC43 (only VFI) in the micromolar range. Plaque assay results were supported by in silico predictions evidencing that both the peptides could interact with sites in SARS-CoV-2 RBD and S2 subunit containing important clusters of highly conserved residues. Another important consideration, which further stimulates the potential use of the two peptides as novel therapeutic agents, is their absent toxicity. Altogether these data highlight the importance of the two tripeptides as the shortest antiviral agents designed and till now tested against SARS-CoV-2 infection. These two peptides could be used also as template for the development of new antivirals against betacoronavirus infection.

## Figures and Tables

**Table 1:** The nucleic sequence ACATTACAC were found in SARS-CoV-2, SARS-CoV-1 and MERS, whereas GTGTTTATT in the genome of SARS-CoV-2 and HCoV-OC43. Starting positions of each sequence are reported and, if not present, this is indicated as “/”.

SEQUENCE	SARS-CoV-2	HCoV-229E	HCoV-OC43	SARS-CoV-1	MERS	HSV-1	EA-71	HIV-1	YFV	WNV	DENV-2
ACATTACAC	25,372	/	/	25,247	25,227	/	/	/	/	/	/
GTGTTTATT	23,463	/	24,582	/	/	/	/	/	/	/	/

## Data Availability

The data presented in this study are available on request from the corresponding author. Authors can confirm that all relevant data are included in the article.

## Supplementary Materials

The following supporting information can be downloaded at: https://www.mdpi.com/article/10.3390/v14102103/s1, Figure S1: LC-MS spectra of peptides; Figure S2: TLH and VFI structure; Figure S3: Molecular simulation of tripeptides interacting with surface proteins of different human viruses; Figure S4: Molecular simulation of the peptide AAA interacting with SARS-CoV-2 spike protein; Figure S5: Antiviral activity of tripeptides against HCoV-229E; Figure S6: BLI interaction analysis with TLH and VFI; Figure S7: BLI interaction analysis with AAA and FIV; Table S1: Cytotoxicity and antiviral activity of tripeptides against representatives of enveloped and naked ssRNA+ (HIV-1, DENV, YFV, WNV, EVA71) and dsDNA (HSV-1) viruses.
