# Supplementary material for: Design of Three Residues Peptides against SARS-CoV-2 Infection
Source: Viruses. 2022 Sep 22;14(10):2103. doi: 10.3390/v14102103 (PMC9612326; doi:10.3390/v14102103)
Supplement: Supplementary file 1 [file viruses-14-02103-s001.zip › viruses-1873540-supplementary.pdf]

**Figure S1.** Spectra of peptides. A) TLH; B) VFI.

# A

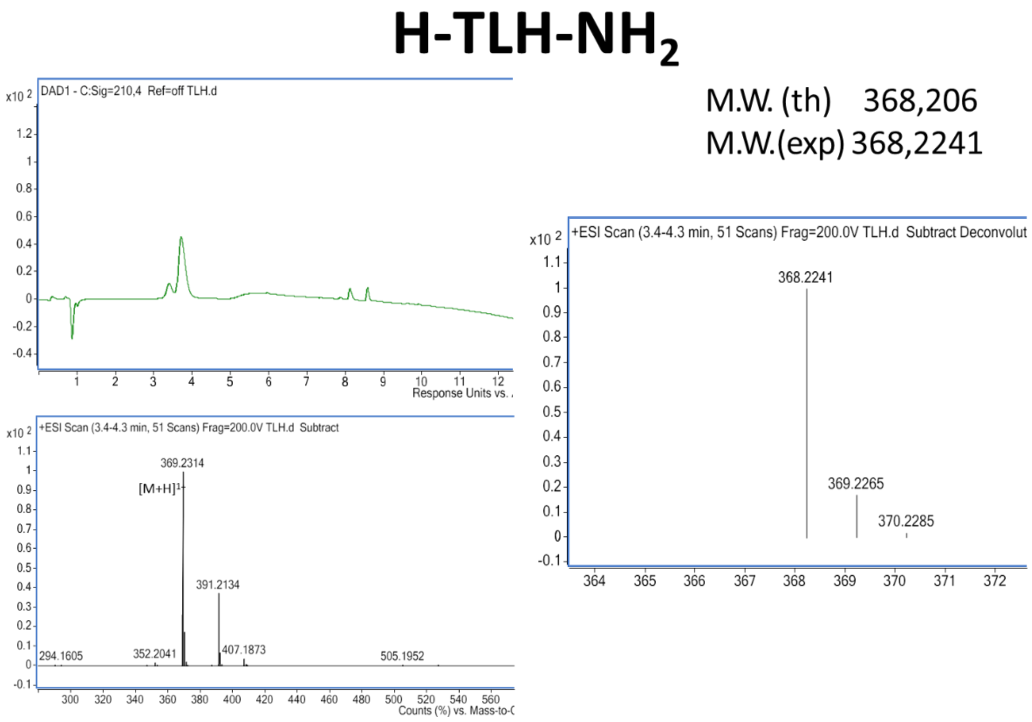


# B

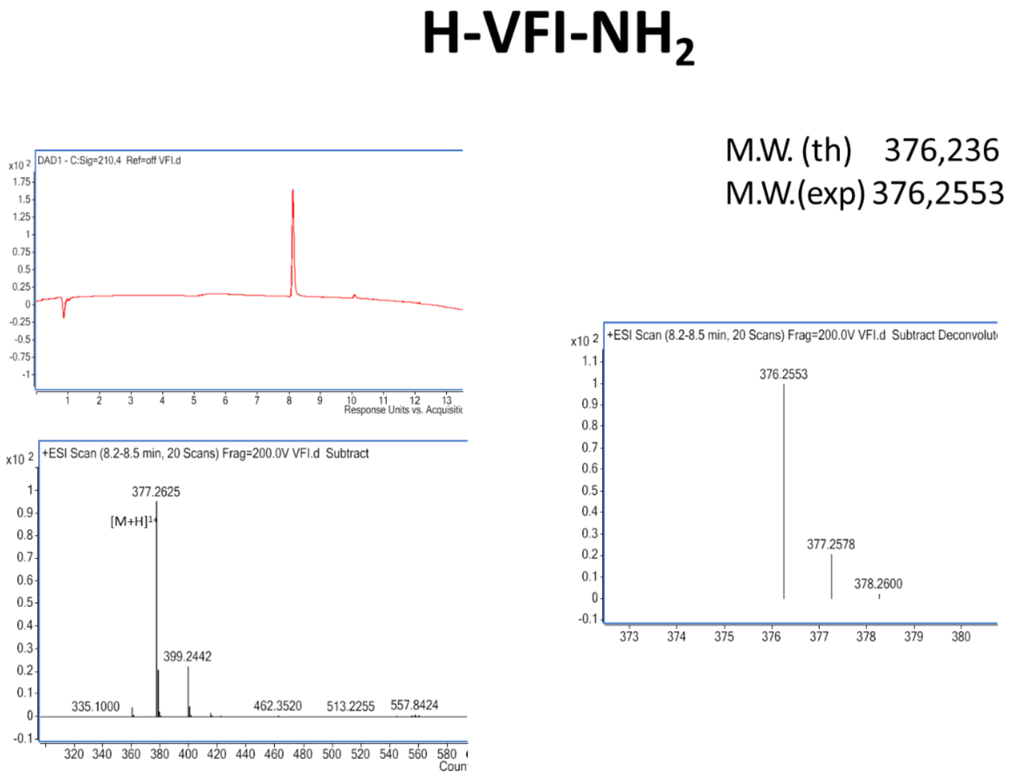

**Figure S2.** TLH and VFI structure. TLH and VFI were indicated with their chemical and structural conformations designed in PyMOL.

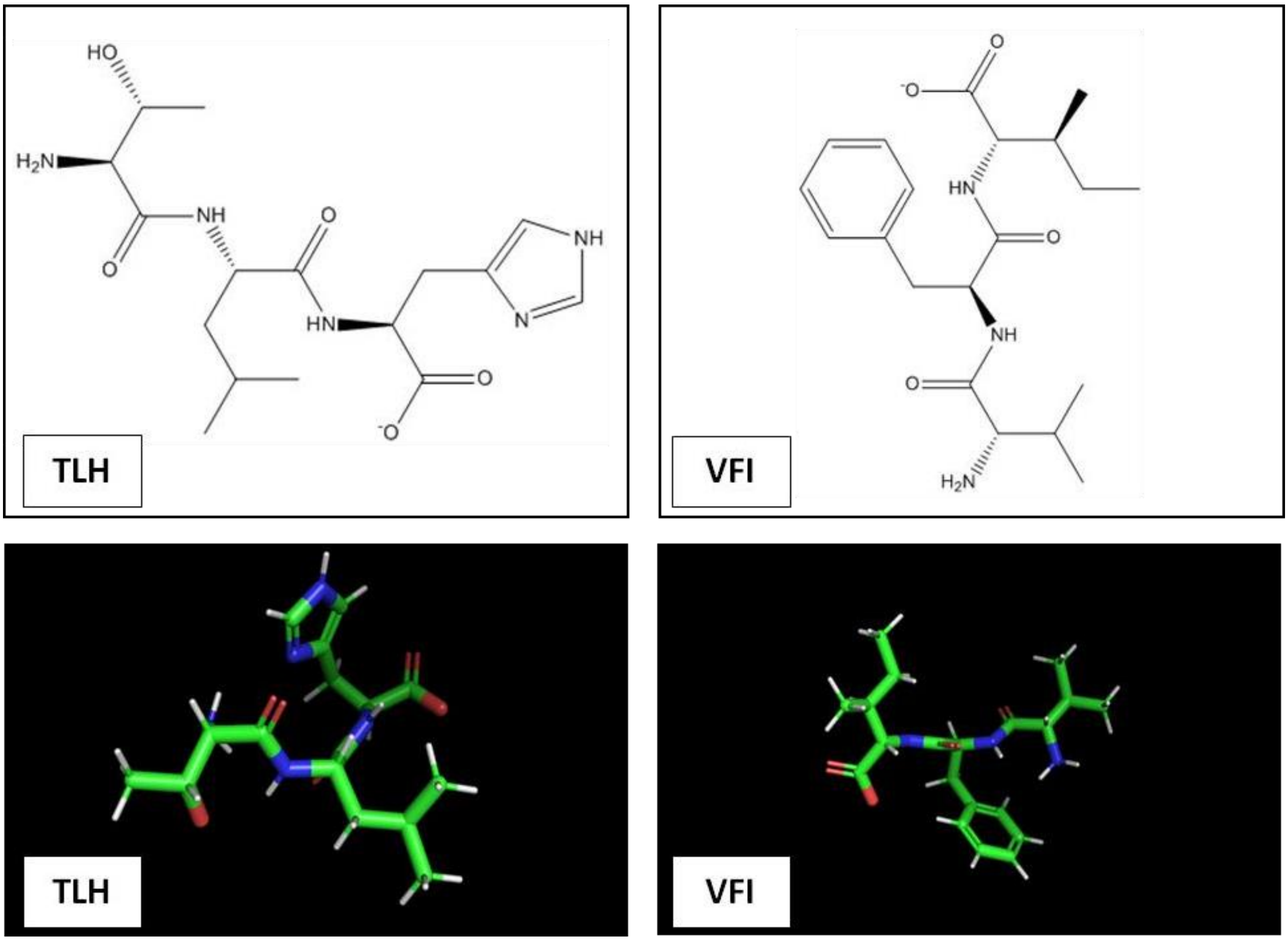


**Figure S3.** Molecular simulation of tripeptides interacting with surface proteins of different human viruses.

**Crystal structure of the spike protein of human coronavirus 229E**
PDB ID: 6U7H

SEQUENCE LENGTH: 1159

| **Spike** – TLH interaction | **Spike** – VFI interaction |
|---|---|
| 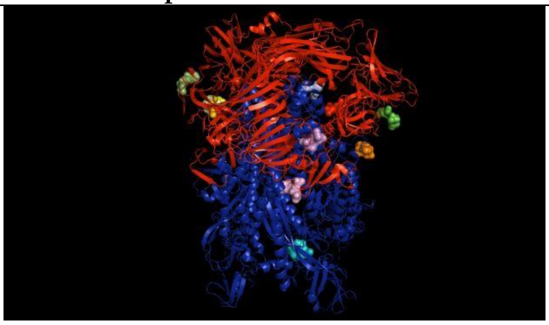 | 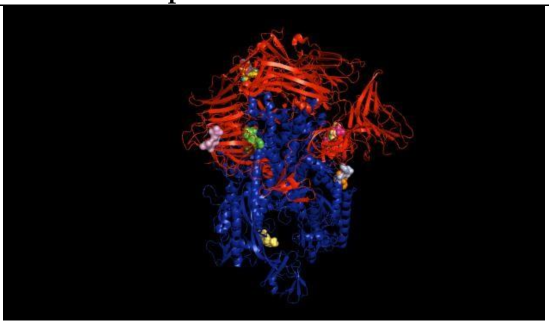 |

| **Rank** | | | | | | | | | | |
|---|---|---|---|---|---|---|---|---|---|---|
| **Docking Score_TLH** | -114.203 | -109.572 | -107.434 | -102.800 | -100.077 | -100.039 | -99.703 | -99.119 | -97.779 | -97.423 |
| **Docking Score_VFI** | -117.192 | -109.467 | -111.095 | -111.753 | -109.428 | -111.392 | -109.293 | -112.190 | -113.759 | -112.871 |

**Crystal structure of the extracellular domain of glycoprotein B from Herpes Simplex Virus type I**
PDB ID: 2GUM

SEQUENCE LENGTH: 628

| **Glycoprotein B** – TLH interaction | | | | | | **Glycoprotein B** – VFI interaction | | | | |
|---|---|---|---|---|---|---|---|---|---|---|
| 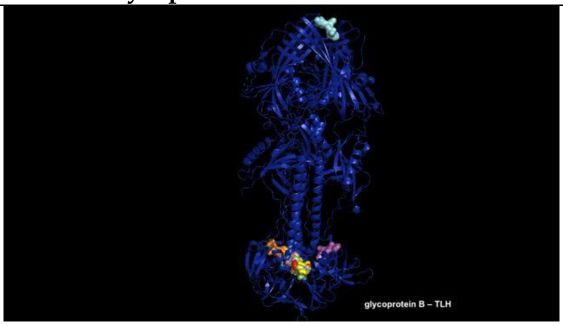  | | | | | | 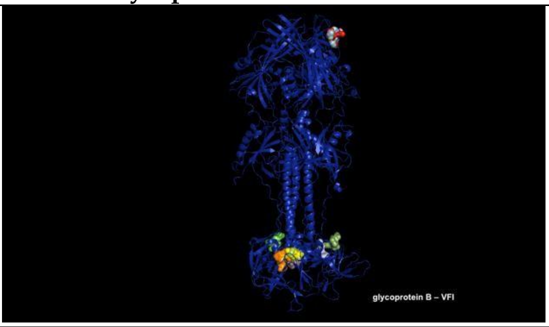  | | | | |
| **Rank** | | | | | | | | | | |
| **Docking Score_TLH** | -115.75 | -111.99 | -111.86 | -108.87 | -107.62 | -107.15 | -107.08 | -106.87 | -106.01 | -105.99 |
| **Docking Score_VFI** | -115.37 | -110.82 | -106.90 | -105.78 | -105.76 | -105.26 | -105.18 | -104.84 | -104.79 | -104.24 |

**Crystal structure of Flavivirus Envelope protein**

PDB ID: 5JHM

SEQUENCE LENGTH: 416

| **Glycoprotein E** – TLH interaction | | | | | | **Glycoprotein E** – VFI interaction | | | | |
|---|---|---|---|---|---|---|---|---|---|---|
| 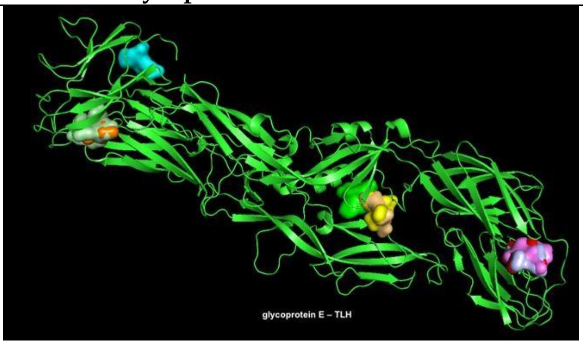  | | | | | | 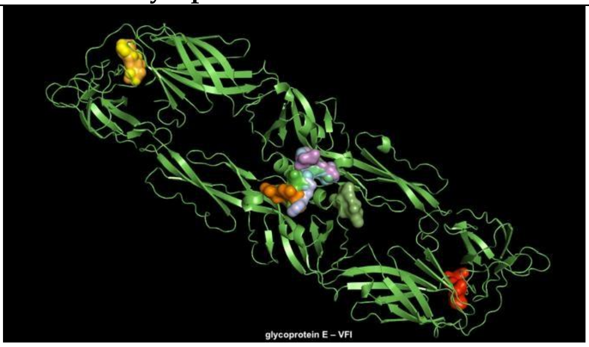  | | | | |
| **Rank** | | | | | | | | | | |
| **Docking Score_TLH** | -106.721 | -105.994 | -104.856 | -97.915 | -97.048 | -97.033 | -96.045 | -95.094 | -94.849 | -93.845 |
| **Docking Score_VFI** | -116.326 | -112.563 | -111.378 | -108.393 | -107.79 | -107.726 | -107.447 | -107.316 | -106.653 | -105.864 |

**Model of gp120 and gp41, including variable regions**

PDB ID: 5JHM

| | green | blue |
|---|---|---|
| **CHAINS** | Envelope glycoprotein gp120 | Envelope glycoprotein gp41 |
| **SEQUENCE LENGTH** | 470 | 140 |

| **Gp120 -** TLH interaction | **Gp120 -** VFI interaction |
|---|---|

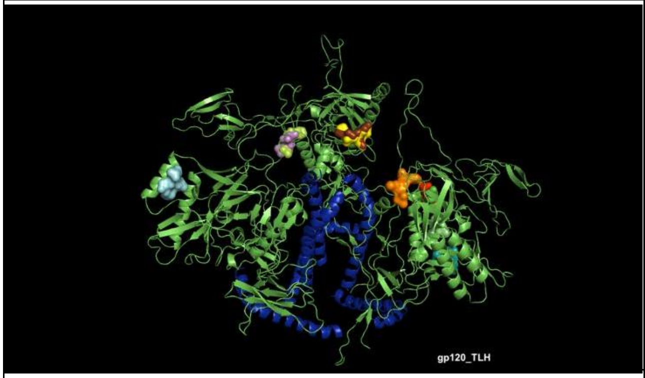


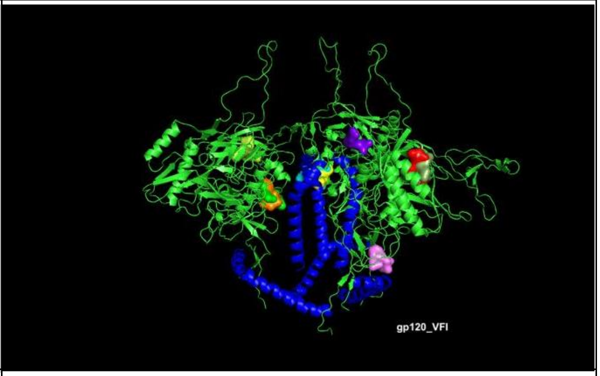


| **Rank** | | | | | | | | | | |
|---|---|---|---|---|---|---|---|---|---|---|
| **Docking Score_TLH** | -113.320 | -111.544 | -105.598 | -102.861 | -101.872 | -101.187 | -101.034 | -100.379 | -99.968 | -99.754 |
| **Docking Score_VFI** | -123.263 | -120.879 | -120.266 | -118.970 | -118.342 | -116.826 | -116.440 | -116.243 | -115.970 | -114.700 |

**Figure S4.** Molecular simulation of the peptide AAA interacting with SARS-CoV-2 spike protein.

| **SARS-CoV-2 Spike** – AAA interaction | |
|---|---|

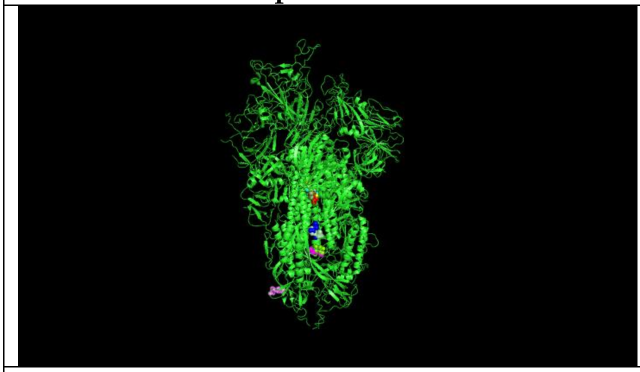

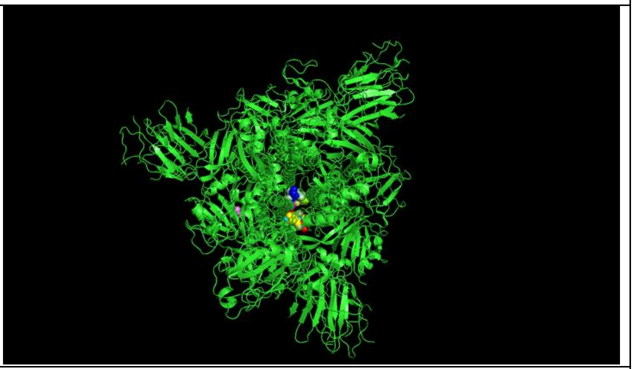

| **Rank** | | | | | | | | | | |
|---|---|---|---|---|---|---|---|---|---|---|
| **Docking Score_TLH** | -74.037 | -70.332 | -68.469 | -67.268 | -66.994 | -65.313 | -65.067 | -63.612 | -63.193 | -62.332 |

**Figure S5.** Antiviral activity of tripeptides against human coronavirus 229E. A) co-treatment; B) virus pre-treatment; C) cell pre-treatment; D) post-treatment. CTRL+ refers to positive control (ivermectin 12 μM) and CTRL – indicates the negative control (only infected cells); *p ≤ 0.0001.

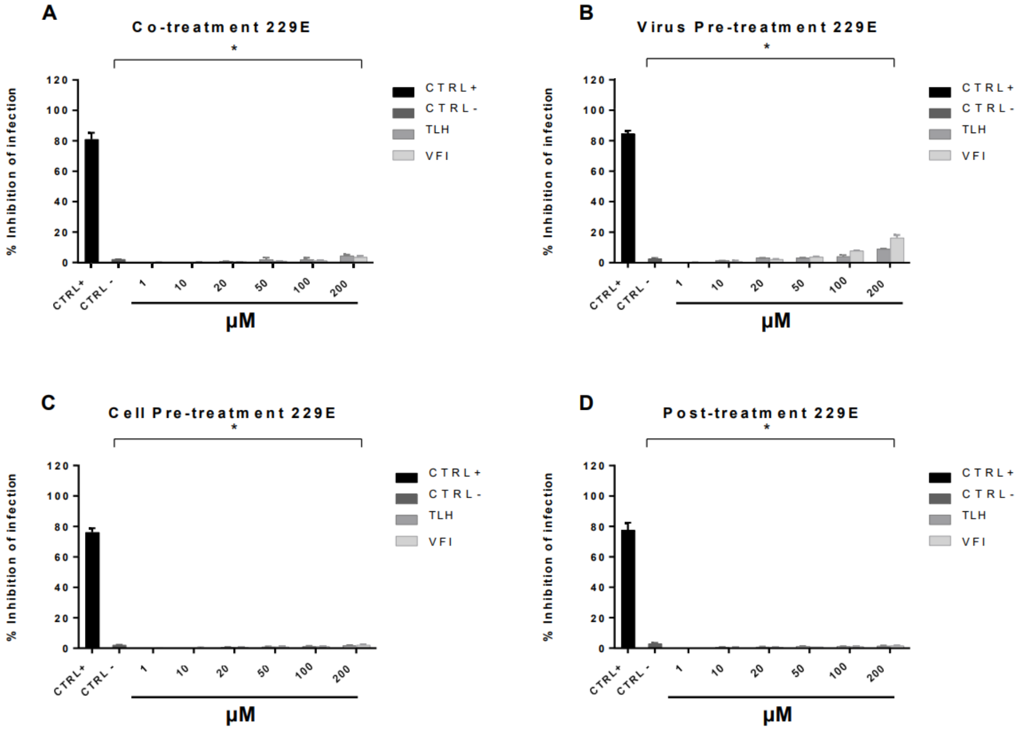


**Figure S6.** BLI interaction analysis with TLH and VFI. RBD (0.2 μM) either alone in solution or premixed with VFI (A) and TLH (B) peptides, tested at different concentrations, and VFI (A) or TLH (B) only, were subject to BLI analysis. BLI was performed at 25°C in PBST (10 mM phosphate, 150 mM NaCl, 0.05% Tween 20, pH 7.4).

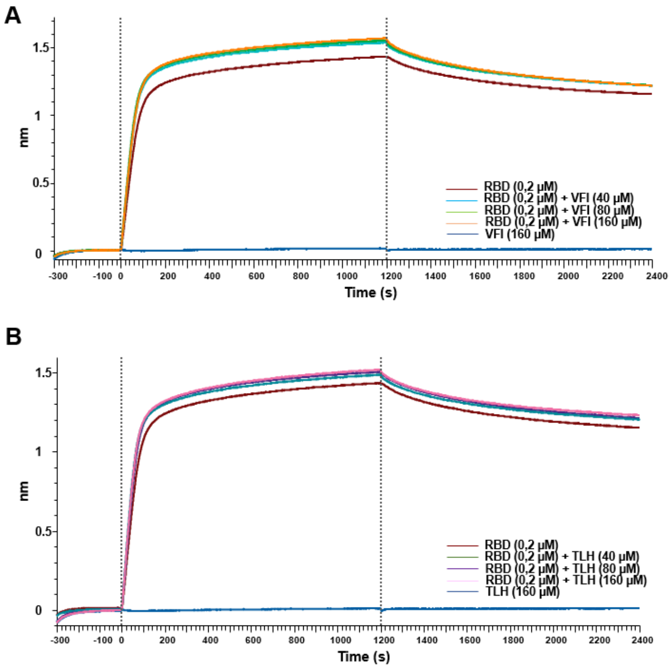

**Figure S7.** BLI interaction analysis with AAA and FIV. RBD (0.2 μM) either alone in solution or premixed with AAA (A) and FIV (B) peptides, tested at different concentrations, were subject to BLI analysis. BLI was performed at 25°C in PBST (10 mM phosphate, 150 mM NaCl, 0.05% Tween 20, pH 7.4). The corresponding plots (C, D) of steady-state binding from the end of the association phases (nm), after the subtraction of RBD signal, against analyte concentration were used to calculate the steady-state affinity by nonlinear regression analysis using Graph-Pad 5 software. KD values cannot be calculated in the experimental conditions used.

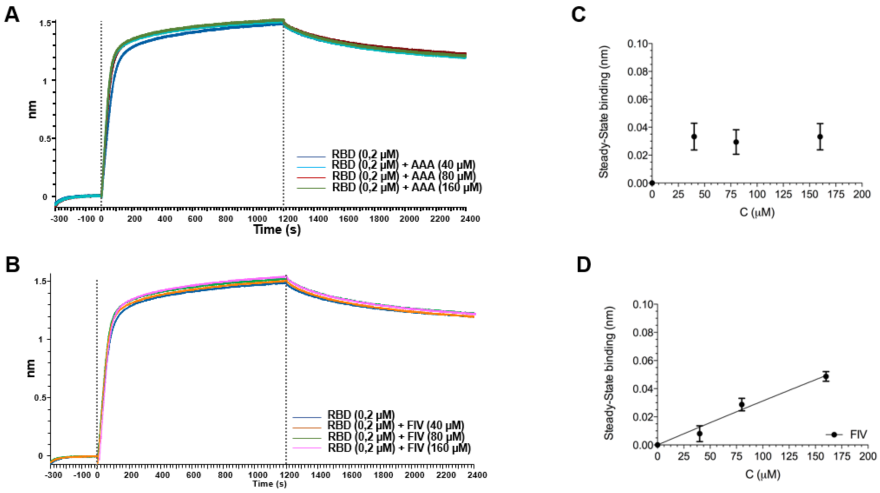


**Supplementary TableS1.** Cytotoxicity and antiviral activity of tripeptides against representatives of enveloped and naked ssRNA$^{+}$ (HIV-1, DENV, YFV, WNV, EVA71) and dsDNA (HSV-1) viruses.

| Compound | MT-4 | HIV-1$_{IIIB}$ | BHK-21 | DENV-2 | WNV | YFV | Vero-76 | EV-A71 | HSV-1 |
|---|---|---|---|---|---|---|---|---|---|
| | $^{a}CC_{50}$ | $^{b}EC_{50}$ | $^{c}CC_{50}$ | $^{d}EC_{50}$ | | | $^{e}CC_{50}$ | $^{f}EC_{50}$ | $^{f}EC_{50}$ |
| VF1 | >200 | >200 | >200 | >200 | >200 | >200 | >200 | >200 | >200 |
| TLH | >200 | >200 | >200 | >200 | >200 | >200 | >200 | >200 | >200 |
| | | | | | | | | | |
| ***Reference Compound** | | | | | | | | | |
| 2'-C-methyl-guanosine | - | - | >100 | 1.2 | 1.7 | 2.6 | - | - | - |
| Azidothymidine | >100 | 0.03 | - | - | - | - | - | - | - |
| 2'-C-methylcytidine | - | - | - | - | - | - | >100 | 2 | 2 |

[a] Compound concentration (μM) required to reduce the proliferation of mock-infected MT-4 cells by 50%, as determined by the MTT method.
[b] Compound concentration (μM) required to achieve 50% protection of MT-4 cells from HIV-1 induced cytopathogenicity, as determined by the MTT method.
[c] Compound concentration (μM) required to reduce the viability of mock-infected BHK cells by 50%, as determined by the MTT method.
[d] Compound concentration (μM) required to achieve 50% protection of BHK cells from respectively DENV-2, WNV, YFV induced cytopathogenicity, as determined by the MTT method.
[e] Compound concentration (μM) required to reduce by 50% the viability of mock-infected Vero-76 cells, as determined by the MTT method after 3 days.
[f] Compound concentration (μM) required to achieve 50% protection of Vero-76 cells from EV-A71 and HSV-1 induced cytopathogenicity, as determined by the MTT method at day 3.
